# Perspectives, Expectations, and Concerns of European Patient Advocates on Advanced Therapy Medicinal Products

**DOI:** 10.3389/fmed.2021.728529

**Published:** 2021-11-23

**Authors:** Stefano Benvenuti, Chiuhui Mary Wang, Simona Borroni

**Affiliations:** ^1^Fondazione Telethon, Milan, Italy; ^2^Gruppo Famiglie DRAVET, Milan, Italy

**Keywords:** gene therapy, cell therapy, patient advocacy, ethical analysis, tissue therapy, patient—centered care, bioethic

## Abstract

This paper presents the results of a qualitative study based on semi-structured interviews of 10 expert patient advocates on several different issues around Advanced Therapy Medicinal Products (ATMPs). The interviews were conducted between February and May 2020 based on a guideline with a list of 8 topics that covered concerns about safety and ethics, access problems and limitations, pricing of ATMPs and educational needs for patient communities. Overall, the interviewees expressed a high degree of convergence of opinions on most of the topics and especially on the identification of the reasons for concern. Conversely, when asked about possible solutions, quite a wide range of solutions were proposed, although with many common points. However, it highlights that the debate is still in its infancy and that there are not yet consolidated positions across the whole community. A general concern emerging from all the interviews is the potential limitation of access to approved ATMPs, both due to the high prices and to the geographical concentration of treatment centers. However, patients recognize the value of a model with a limited number of specialized clinical centers administering these therapies. On the ethical side, patients do not show particular concern as long as ATMPs and the underlying technology is used to treat severe diseases. Finally, patients are asking for both more education on ATMPs as well as for a more continuous involvement of patient representatives in the whole “life-cycle” of a new ATMP, from the development phase to the authorization, from the definition of the reimbursement scheme to the collection of Real Word Data on safety and long-term efficacy of the treatment.

## Introduction

New scientific progress in cellular and molecular biotechnology has led to a new field of biomedicine which offers new opportunities for the treatment of diseases and dysfunctions of the human body (1–6). Advanced therapy medicinal products (ATMPs) are the treatments resulting from the advancement in this new field and they ≪may be used in or administered to human beings with a view to restoring, correcting, or modifying physiological functions by exerting principally a pharmacological, immunological or metabolic action≫ (7, 8). According to the European Regulation (EC) No 1394/2007 (7) and the Directive 2001/83/EC (8) ATMPs are medicines for human use that are based on genes, tissues or cells; a similar definition, although not identical, is adopted by the United States Food and Drug Administration (FDA) (9).

Despite the large number of ATMPs in development (10), currently, only 20 ATMPs have been approved by the FDA (11) and even fewer by the European Medicines Agency (EMA). The products under development are intended to treat a wide variety of conditions, spanning cancers, inherited diseases, and other chronic conditions (10).

A number of technical challenges are still open, both in the development of such products (12–17) as well as in the pricing and access to these therapies, including ethical and economic issues, follow up management, logistic and delivery issues, and equity of access (18–22). Expert patient advocates could provide important insights into these discussions, especially on ethical and access issues (23–31). Patient perspectives are crucial, especially when considering the need to define the real value of these therapies in terms of improvement for quality of life (QoL) and thus, a fair price (30, 31). Patient contribution in the collection of follow-up data is also crucial to provide information on the effectiveness of the therapies on a long-term and real-world basis (32, 33).

In addition, specific inputs from patients are needed on organization of healthcare system and clinical centers for the delivery of ATMPs as well as organization of clinical trials operation (26, 28). These inputs are specifically useful to contribute to the definition of the best ATMP delivery model to manage cross-border mobility issues and facilitate patients' access to therapies.

For these reasons, it is paramount that academia, industries, health systems, regulatory, Health Technology Assessment (HTA) bodies and payers involve patients in the processes of research, development, approval, pricing and marketing of ATMPs (28).

For the adoption of ATMPs as a part of treatment plans, patient empowerment will be essential. Educational activities on specific topics will be fundamental to enable patients to approach ATMPs with realistic expectations of the risks, potential benefits and to participate in clinical trials with increased awareness and the necessary basic knowledge to take informed decisions (30, 34, 35).

In this paper, we have gathered perspectives from patient advocates on a number of different issues, such as relevance for patients of a discussion on ATMPs, barriers and solutions to improve access to specialized centers, pricing, feasibility, and relevance of the collection of long-term follow-up data. Also included were particularly sensitive issues such as equity of access, safety and ethical concerns and the value a patient recognizes for a treatment that might change the course of the disease, including issues related to the sustainability of national health systems.

## Methods

### Interviewees

Interviewees were selected based on their proven track of patient advocacy, reputation, geographical area, and disease area representations. In addition, they should represent umbrella patient organizations, single patient organizations, independent experts, or is a patient themselves. Patient advocates who had undergone training on medicine research and development or patient academies were preferred. Patient advocates are appropriate interviewees because they are informed and are visionary trend setters for their disease areas. This makes the idea for interview with respect to the general patient population. Ten patient advocates were selected and invited for interview for their opinions and qualitative study. The interviewees located in different geographical areas within Europe (Belgium, Germany, Ireland, Italy, Netherlands, Poland, Spain, Sweden) and several disease areas (cancer, rare disease, multiple sclerosis, diabetes, Parkinson's disease, hemophilia). Whether an ATMP was available or not for their diseases of interests was not taken into consideration for the selection. With the explicit agreement of all interviewees, a full list of their names and affiliation is included in the Acknowledgments. No compensation was foreseen for the interviewees.

### Interview Guide and Methodology

The methodology for the interviews was developed based on standards for qualitative research (36, 37). The interview guide was developed within the project RESTORE, a European Commission funded project (Grant Agreement number: 820292). A guideline for the interview with a list of 8 topics was developed and piloted in 2 mock interviews with Italian patient advocates. The responses of the mock interviews are not included in the results. The interview covered the following topics: (1) Relevance for the patients of a discussion about ATMPs; (2) Barriers and solutions to improve patient access to specialized centers qualified to administer ATMPs; (3) Pricing; (4) Feasibility and relevance of the collection of long-term follow-up data after treatment; (5) Access pathways; (6) Safety concerns; (7) Ethical concerns; (8) Education and training needs for patients. This interview guide was sent to the interviewee together with the consent information sheet before every interview (Supplementary Materials). The guidelines were shared beforehand to facilitate the conversation during the interview and reduce any possible anxiety in interviewees who may otherwise have felt under examination.

### Data Collection

The interviews were conducted between February and May 2020. Each interview lasted 30–45 min and was recorded. Before starting the interview and the recording, the interviewer presented the main points of the consent sheet. Specific emphasis was put on the fact that the interviewee can skip any question/topic and stop the interview at any time. Most of the interviews were run by an expert patient advocate (co-author of this report) to make interviewees feel comfortable being in a conversation with a peer.

The transcript of the recording was sent to the interviewee for cross-check, validation and to avoid unintended bias by the interviewers. The recordings are kept as confidential and any opinion expressed during the interviews is reported under the so-called Chatham House Rule (38). Quotations from specific interviews are included in the results without references to the interviewee who expressed them to preserve the confidentiality of the opinions expressed.

## Results

The results are presented in 8 paragraphs following the structure of the interview guidelines:

### Relevance for the Patients of a Discussion on ATMPs

Almost all those interviewed (9/10) agreed that ATMPs are a “hot topic” for patient communities.

Some of them underlined that this is a “hot topic” especially for:

- Specific disease areas, for instance blood disorders, cancers and diseases with genetic origin / rare diseases.
- Diseases where, currently, there are no treatments available.

For diseases where treatments are available, even if the disease is chronic and lifelong, if persons are able to have a good quality of life, there is less excitement about ATMPs than in diseases where this is not the case.

In addition, according to some of the interviewees, the interest in advanced therapies is due to the fact that they may target the root causes of the diseases. Thus, for some disease areas there is the expectation that a curative solution might be possible. The large number of currently ongoing clinical trials with cell and gene therapies has attracted the interest of patients. They are especially keen to learn more about these advanced therapies; how they work, how patients will benefit from them and when they will be accessible.

Almost half of the interviewees (4/10) agreed about the importance of offering patients good quality and targeted communication about ATMPs, especially on:

- The mechanisms of action of gene therapy and of the adopted viral vector.
- The benefits and risks, focusing also on the potential side effects (safety).
- A better understanding of which diseases could potentially be treated with ATMPs in order to avoid high and unrealistic patient expectations.

### Barriers and Solutions to Improve Patient Access to Specialized Centers Qualified to Administer ATMPs

Eight out of ten interviewees think that the creation or recognition of specialized clinical centers is the best model for delivering ATMPs. The major reasons that lead to the preference for this model are the following:

- treating a greater number of patients will allow the centers to gain experience in administering ATMPs but also in the management of the possible toxicity of these therapies that might put patients at risk;
- in smaller centers with fewer patients treated and consequently less expertise, some side-effects may be missed or treated in such a way that it might negatively affects the final treatment outcome;
- in a model with several centers in every country, geographically distributed and treating a relatively low number of patients, some concerns may arise about inconsistency in treatment delivery, in monitoring treatment response over time and the difficulty of sharing data;
- the centralization of real-world data is crucial for the success of ATMPs, not just scientifically, but also clinically. Due to the initially small number of patients likely to be treated, it will be imperative that data is shared to obtain maximum benefit for the majority of patients. One of the main concerns raised regarding data sharing was indeed the risk that valuable data might be collected in isolation without sharing between other hospitals and principal investigators;
- the delivery of some of these therapies require special devices, specific clinical settings and the certification of hospitals to administer the treatment;
- having specialized centers is beneficial for patients themselves, for clinicians, regulatory authorities and pricing and reimbursement authorities who can rely on a form of quality certification that ensures uniformity and quality of the treatment.

Few interviewees (2/10) highlighted the fact that ATMPs could increase inequality in a patient population as the need to administer the treatment in a limited number of specialized centers will constitute an additional barrier to access. Not every patient can travel to get treated, therefore some patients in certain areas might not be able to reach the centers of excellence and thus will not have access to the treatment they need. From a citizens' perspective, this is not acceptable.

The common opinion is that these therapies (or at least some of them) need to be centralized in experts centers at EU level but there is also a need to create a support system that allows patients to access those centers no matter where they live.

Interestingly, two of the interviewees suggested pricing ATMPs as a service. According to this model, industries should consider providing a full service, including both the drug and the clinical costs for its administration as well as paying attention to include patients from different locations in the marketing plans. They also need to quantify how these products are increasing the QoL of the patients in a much broader way. In the end, everything should be revaluated considering ATMPs as a service not simply as a drug.

### Pricing of ATMPs

The majority of interviewees (7/10) agreed that high prices of ATMPs are an issue and, in some cases, could be an obstacle for access to the therapy. The main concern is that high pricing leads to prolonged discussion and negotiation with Payers on reimbursement, slowing down access for patients. Some interviewees (5/10) also highlighted that in the long run, high prices could threaten the sustainability of national health care systems.

Almost half of the interviewees (4/10) report that there should be transparency on how prices are set, on the components to be assessed and on how the incentives are defined. The lack of clarity in the definition of prices could lead to inequalities between the prices of ATMPs and the prices of other lifesaving health treatments such as surgery. They recognize pricing is a complex process and believe both costs and value should be taken into consideration. The price may be linked to the costs of the therapies and to the incentives received by the developers, and even more importantly the need to focus on the value of the treatments. The definition of value should include not only the direct benefit of the treatment but also the cost saving due to the effect of that treatment on the progress of the disease and, consequently, on the burden of the disease.

Most agree that the discussion on the price of treatments should be done in a framework considering all the costs needed to deliver the treatment to patients, meaning costs of follow up and clinical data registry, screening, organizational costs to deliver the therapy to patients (professionals, specialized nurses, and special settings) and any other additional cost to get the real cost of the treatment for patients.

Interviewees expressed different opinions commenting on the innovative agreements recently signed between industries and payers to enable access to ATMPs. These innovative agreements include several different schemes designed to find a balance between the high cost of ATMPs and the uncertainty on their efficacy, including their long-lasting effectiveness; such schemes are generally referred to as Outcome Based Managed Entry Agreement, Risk Sharing Agreements, Value based price and Delay payment (20, 39–41). Managed entry agreements can facilitate patient access to treatments and are especially important for life-saving treatments where patients with high unmet needs should have early access. In many other cases, Managed Entry Agreements do not solve the issue of high prices in the long term. In addition, it may prove difficult for the competent authorities to apply them, especially for those new treatments where it could be difficult to evaluate the efficacy over a long-term period. For value-based price the definition of “value” may be controversial. The current limitation is that, so far, nobody can really say whether and for how long a treatment is going to work. Finally, delayed payments are not considered by the interviewees as a solution for high prices as the payment, sooner or later, will still impact heavily on healthcare budget.

According to the interviewees, the most promising instrument that has not yet been frequently used is pooling procurement among countries; a greater number of patients would give competent authorities a higher bargaining power when discussing the price.

When value-based price was discussed, interviewees were asked what “value” means for them. Most interviewees indicated that, for them, value in this context means the value for patients, which should be assessed based on data provided by patients: improvement in quality of life and safety. Patients themselves should be involved in providing data as well as in defining what should be intended with “value for patients.” This means spelling-out the reasons why a certain product should be reimbursed and what benefit it could provide to the patient community. This value should be strictly linked with the improvement in the quality of life of patients. In addition, for most ATMPs, long-term follow-up assessing the safety of the product is recommended. For all the above-mentioned reasons, patients should work with Payers and HTA Agencies, with the final aim of contributing to the definition of standards in outcome measures and to the set-up of post-marketing registries.

Moving back to the development phase of the drug, value is defined as the measurable benefit during clinical trials. A strong statement by most of the interviewees is that, where possible, the endpoints of clinical trials should focus on the patient and not on the product. This means, for example, setting up a single platform for controlled clinical trials on a specific disease to evaluate and directly compare in the same trial different compounds from different companies. This will allow the direct evaluation of the value of each single treatment and the real benefit for patients.

### Feasibility and Relevance of the Collection of Long-Term Follow-Up Data After Treatment With ATMPs

Interviewees suggest that patients tend to be generous in giving their data, also after the treatment, for the sake of research and for the benefit of other patients. However, half of the responders (4/8) indicated that it could indeed be difficult to involve patients in data collection for long-term follow up. All agreed that engagement of patients and their family is a critical factor for the success of long-term data collection.

Interestingly, some of the responders (3/8) suggested that a possible way to involve more patients in providing long term follow up data, could be to involve patient representatives in defining patient reported outcome measures (PROMs) and the questionnaires often associated with long-term follow-up and the assessment of patients' quality of life. In their opinion, patients will generally be more willing to answer questions that are meaningful to them.

Thus, it is important to develop together with patients a set of measures, asking fewer questions but that are more relevant to them and closer to their unmet needs in daily life. Having patient representatives involved in the design and definition of PROMs could also provide the scientific community and the regulators relevant insights on the most pressing unmet needs for the patient community.

Another point of view is that patients need to be motivated to share their data: patient engagement should be seen as a two-way exchange where patients provide value (data) and receive value in return. Therefore, it is necessary to determine how to give something back to patients in order to demonstrate that the investment of their time is worthwhile. On this point, interviewees suggested that patients should be provided with information both at a cohort level but also at individual level to be able to calculate how far they are from “the mean.” Knowing to which percentile a patient belongs could empower them to either better accept the condition or to take action and look for further therapeutic strategies that could improve their quality of life. Another possibility would be to lower the cost of treatments for those who are engaged and compliant in data collection. Finally, it is crucial to give feedback to patients about how the data are used. Some of the respondents suggested that to reduce possible concerns and encourage patients to share their data, good supervision and a good data management framework is necessary. This includes transparency about the use of data, who is going to use them and how patients can withdraw their consent in the event they no longer want to share their data. Information should be given about servers where the data are stored and their compliance with the European General Data Protection Regulation (GDPR).

Patient organizations could play a role in preventing patients dropping out of long-term post-treatment data collection by providing education about the importance of having the data to demonstrate the value of the treatments. It should also be explained to patients that lack of data about the value of the treatments could lead to later access to the therapies. Another point raised by one of the interviewees is the role that patient organizations could and should have in collecting data. This interviewee considers it very important that patient organizations act as the preferred channel to link patients and the competent authorities. This will ensure that patients reach competent authorities without any filter by individual clinicians or by the industry. Information is of value to citizens and in the current times, data are becoming a new currency.

Most of the respondents recognized that there are no longer technical barriers to the engagement of patients; data can be provided remotely and thus, it is no longer necessary to go back to clinical centers for every follow-up data collection point. Data collection can be done by remote monitoring, via mobile apps or organizing conference calls with patients at home. Upon direct questioning about data sharing, 6 out of 7 respondents agreed that patients are more likely to be willing to share their data than they are to be concerned about it. This is especially the case in extremely rare diseases, where patients hope that sharing their data could stimulate researchers to start studying their diseases and eventually improve their condition.

Concerning data collection, some additional interesting ideas are long-term and stable data collection not linked to a specific product but rather to a condition or a group of diseases, which could help in better assessment of the standard of care and its value as well as to establish a baseline for the assessment of old and new products. To facilitate that process, with the contributions of different pharma companies, national funds dedicated to set up and maintain the registries needed for pharmacovigilance should be created.

### Access Pathways for ATMPs

The interviewees were asked about the appropriateness and duration of the processes for the approval of Clinical Trials, for Marketing Authorization and for price negotiation of ATMPs. Almost all the interviewees (8/10) thought that the process required for getting a treatment on the market takes too long.

Three interviewees suggested that the approval process should differentiate according to:

- The different nature of the therapies: autologous cells therapies versus “off the shelf” products. For example, autologous cell therapies are considered less risky for patients, thus the process could be faster.
- The different disease areas: high prevalence diseases vs. rare and complex diseases and diseases where there are treatments vs. diseases where there are not.

With regard to risk assessment in the approval process, it should be considered that this process has been developed for high prevalence diseases, therefore not considering that, in rare diseases, where the condition is severe and debilitating, the risk that patients are willing to take is higher.

While safety considerations are paramount for some of the interviewees (4/10), another suggested that the concept of acceptable risk, as well as safety, should be reconsidered taking into account the specific disease or patient situation. In accordance with this last comment, the risk assessment in the approval process should be reviewed because, in severe and life-threatening diseases, the level of risk that patients are willing to take is higher than in diseases where an alternative therapeutic option is available. For this reason, a different framework of approval for different diseases with different unmet needs should be created. As an example, one of the interviewees mentioned the “Right to try” model, signed into US law May 30, 2018. This could be helpful to patients who have been diagnosed with life-threatening diseases or conditions, who have tried all approved treatment options and who are unable to participate in a clinical trial. The combination of these conditions should allow them to access unapproved treatments that have completed Phase I.

With respect to the price negotiation process, the majority of the interviewees (6/10) agree that it should be improved and accelerated, while a few of them (3/10) commented about the fact that EMA is already taking action in order to accelerate approval processes—i.e., conditional approval, Prime medicine, etc. One of the possible actions suggested by the interviewees to accelerate the access to treatments, is to provide immediate access for certain patients and pre-file a price that can then be corrected after the negotiation. These are the key concepts of an early access scheme already used in France and called the Authorization of Temporary Usage (ATU) (42).

With respect to the speed of access to treatments, considering the limited number of patients included in the clinical trials, some of the interviewees mentioned the importance of having a robust system for the collection of long-term follow up data. This will allow for combining the need for patients to get access to a hopefully life-saving treatment and the need for additional data to evaluate whether that treatment is really providing a significatively higher therapeutic value. On one side the involvement of academia and a system of supranational cooperation have been mentioned as possible way to set up this system for real word data collections; on the other hand, it needs to set up *ad hoc* committees providing ongoing reviews of ATMPs already on the market to look at how efficacious the treatments are in the long term and in the real word setting. In the view of respondents, these committees should include patients, clinicians, regulatory agencies, HTA experts and payers.

### Safety and Concerns on Unauthorized Treatments

Interviewees were consulted on patients' view on all the different unauthorized treatments available on the market. In this question, unauthorized treatments refers to treatments offered outside any legally authorized frame; therefore, for the purpose of this question, EMA authorized products as well as investigational products in an authorized clinical trials or administered under compassionate use were all considered “authorized” (43).

In general, all the interviewees agree about the fact that, especially for life threatening diseases that have no treatment options, patients are more willing to try any sort of treatment, overcoming fears about adverse events. In these cases, patients may be more likely try to obtain any available treatment, even if not authorized, and are willing to pay out of pocket for it.

One of the interviewees also reported that the long waiting time between EMA authorization of the product and its availability for patients due to long HTA and price negotiation procedures, might, in some cases, be one of the causes that push patients toward looking for unauthorized treatments.

Among the respondents, there are different opinions about how to counteract the diffusion of unauthorized treatment. More than half (5/8) think that patients should be better educated about ATMPs. More specifically, proper information is needed about what is on the market and what, if any, are the alternatives. In addition, patients need to know more about the long and complicated path of medicine development and how important it is to be treated in approved centers. According to one of the respondents, patient organizations should also work to empower the patients who may not quite understand the science, to weigh up the risk before going for any treatment they can find. Others (3/8) highlighted that local governments should take actions to counteract false information while, at European level, common rules are needed to sanction those who administer unauthorized treatments.

### Ethical Concerns

Half of the interviewees agrees that there should not be any ethical barrier around therapies that alter the gene without transmitting this modification to the germinal line. A common opinion is that debate on ethical concerns is still at a very early stage. What is strongly affirmed by all interviewees is that all the relevant stakeholders, including patients, should be involved in all discussions about ethical limits. In this respect interviewees highlighted the importance of influencing the public debate on ethical issues to shift the focus toward health benefit for patients, as, according to them, this is the most relevant topic.

In addition, interviewees highlighted the need for more information about what new techniques such as gene editing can and cannot achieve, what the consequences could be and what are the hypotheses. There is a common feeling that it is not currently possible to define the real limits of these technologies, but it is important to determine how the application of these new techniques could change the course of certain diseases. Interviewees agree about limiting the treatments to the cure of genetic disorders avoiding any attempt to modify other physiological characteristics (e.g., eye color, height, etc.).

Finally, some respondents would like a common position to be elucidated on what happens if other less regulated countries develop and make gene editing techniques available to patients before they become available in Europe. Would this lead to European patients traveling abroad for curative treatments?

### Education and Training Needs for Patients

All the interviewees agree on the need for educational tools for patients. Half of them (5/10) also mentioned the need to educate professionals, such as general practitioners, specialists and pediatricians. Only a few (2/10) mentioned the need to educate the general population and interestingly the need to educate policy makers (reimbursement agencies). According to two of the interviewees, the education actions should be addressed to people who are interested in learning about these therapies and should contain only topics that are directly relevant to that audience. In general, they underlined the need for more well-trained expert patients to be involved in development and marketing pathways of medicinal products. Consequently, there is the need for comprehensive training for expert patients in medicine development, approval, reimbursement and HTA. This is essential.

There are two major training focuses identified by the interviewees:

- Train patients specifically on ATMPs, explaining:
  - The differences between ATMPs and other medicinal products currently in use.
  - The differences among the different classes of ATMPs. This means for example explaining the difference between gene therapy with adeno virus and CAR-T cell therapies. Trainees need to understand the specificity of each class of ATMPs in order to understand that not all technologies can be applied to all diseases.
  - The biological mechanism of how ATMPs work in our bodies and what they change, what these therapies are for, who can benefit, and how people have already benefitted.
- Train patients on general research and development processes, similar to the European Patients' Academy on Therapeutic Innovation (EUPATI) training (44). Specifically:
  - How medicines are developed, approved and reimbursed. This includes both the European legislation on the development and approval of ATMPs as well as the national legislation to understand how local authorities make their decisions.
  - How clinical trials are performed, how evidence is collected and why it is important to collect that evidence. Sometimes patients do not have a clear perception of how long it takes to develop a new medicine, therefore it should be explained in order for them to understand why so much time is needed.
  - What are clinical trials?
  - Why is safety so important?

According to the interviewees, to be effective, education should be tailored to the situation of the patient and their interests. Considering the landscape of research on their disease, patients may have different expectations; therefore, to meet those expectations the focus of the educational path has to be wide. For example, patients can be interested because a therapy is coming to the market and they want to know how this therapy works, how it will be administered, what the outcome may be. Other patients who are waiting for a therapy that is not yet on the market may be more interested in how to get access to it before the marketing authorization process is complete. Others, affected by diseases for which there is no research ongoing, may want to know how to stimulate researchers' interest.

Moreover, there is a need to improve the dialogue between patients and clinicians, in order to consider all the possible questions related to new treatments. For patients, to have the possibility to discuss new treatments in a room with other patients and clinicians could be a great improvement and could help make them more comfortable with their decisions. The ultimate goal of a good education program should be to ensure patients properly understand the value out of these products and are in the position to make well informed decisions.

According to the interviewees, the most effective tools to train patients are webinars and face to face meetings, the latter being preferable. Face to face meetings are the preferred tool as they stimulate better exchange among participants and often better absorption of information. As a result, participants in face to face meetings are often better able to transfer their knowledge to other patients, thereby amplifying the effect of the original educational event. Training materials on the web could be an option but are considered less effective. Patient organizations can play a big role in covering these educational needs. They can organize workshops and communication campaigns about ATMPs and they have the capacity to reach a higher number of patients.

## Conclusion and Discussion

This study reports a qualitative description on the expectation and perspectives of ten European patient advocates on ATMPs. Patients advocates recognize that the model of a limited number of expert centers administering ATMPs is the best model for effectively and safely delivering such treatments to patients. From their perspective, the success of the therapy is far more important than the location of the center administering it. In addition, that model will facilitate a centralized collection of data, which is essential to improve both the technology and to generate Real World Evidence (RWE). However, this model for administering ATMPs increases the risk of inequalities in patient access to treatment. To avoid this, ATMPs should be marketed as full-package services, including not only the drug itself but also all the necessary clinical and non-clinical services.

A major barrier preventing patient access to therapy is the very high prices ATMPs are currently marketed at. Patients are fully aware of the threat to the sustainability of health systems posed by such high prices, but this should not limit the access to life-saving treatments. Consequently, patients suggest: (1) a more transparent process for the definition of prices of ATMPs; (2) More support for academia as a possible way to develop less expensive ATMPs; (3) Centralized procurement at EU level to increase the bargaining power, especially for rare conditions and smaller countries. Price negotiation should focus on the concept of value, based on data provided by patients on the improvement of their Quality of Life, measurable benefit during clinical trials in comparison with existing therapeutic alternatives (where available) and measurement of the burden of the disease, meaning the impact of the diseases, in terms of direct and indirect costs. Patient contribution to define the value of the treatment can cover different areas:

1. Efficacy
2. Assessment of the setting, i.e., formulation of therapy
3. Impact on daily life and on Quality of Life. Patients have important insights into disease progression.

Moreover, considering the low number of patients generally included in ATMP clinical trials, it is of utmost importance to have a robust system to collect Real Word Data in long-term follow up. Patients are willing to contribute to Real Word Data collection, however the engagement and empowerment of patient communities is essential to ensure the sustainability of data collection in long-term follow up studies. In addition, to further facilitate patient engagement in the collection of follow up data, they should participate in defining Patients Reported Outcomes (PROs) and in identifying the questions most relevant to them. Patient input into ATMP development is of utmost relevance considering that these are disease modifying therapies. With the aim of accelerating access to treatment, the price negotiation process should be improved and accelerated to guarantee patients early access to innovative therapies. This acceleration despite the probable lack of robust data from clinical trials should be balanced by continuous reviewing of the price and access conditions based on the assessment of long-term Real-World Evidence. Furthermore, the risk assessment during the approval process should be reviewed, taking into account the different nature of the therapies, in particular autologous cells therapies vs. “off the shelf” products. With regard to autologous cell therapies, considering they are considered less risky for the patient, the approval process could be faster. Another key factor that should affect the timing of approval is the disease area and re-evaluating the assessment process taking into account the severity of the disease: in life threatening diseases, the level of risk that patients are willing to take is much higher than in high prevalence disease where some treatments are already available.

Concerning ethical aspects, two key messages are expressed by patients: first, all the relevant stakeholders, including patient representatives, should participate in all discussions about ethical limits and secondly, the public debate should focus more on the health benefit of these therapies. Although the patient voice is being included in ethical debates regarding genome editing (45), inclusion of patient perspectives is not yet carried out systematically. The second request from patients, to focus the ethical debate on health benefit, is especially significant as until now, the debate on ethical aspects of ATMPs has focused almost exclusively on the risk of human enhancement or on the morality of the use of embryonic cells. Thus, a broader more balanced discussion with multiple stakeholders on the ethics of ATMPs is required (44–48). A broader, balanced, discussion on ethical aspects should be envisioned with multiple stakeholders.

One of the key aspects highlighted by the interviewees is the need to manage high and unrealistic expectations of patients. This finding is consistent with the literature on the topic (49, 50) and suggests that additional effort should be devoted to patient education on the general concepts around drug development and on the specific risks related to ATMPs. It is worth noticing that the pros and cons of every new technique are detailed, described and debated in international scientific journals, however, this information struggles to reach patients.

One of the risks which is very clearly perceived by expert patients is the spreading of non-authorized treatments that is reflected in the increasingly frequent crowdfunding requests by patients trying themselves to pay for such treatments. An approach suggested by interviewees is to educate patients on the different technologies under development, explaining their potential but also the associated risk and, finally, the different officially recognized paths to obtain access (clinical trials, compassionate use, approved drugs).

## Limitations

The study was conducted on a very limited sample without any specific sampling strategy aiming at minimizing any potential sampling bias. Although the purpose of the study was to collect the view of patient advocates, this limitation in the sample may limit the validity of the results. In all cases the results presented in this paper should not be considered as the perspective of patient community as a whole. A qualitative approach was selected for this study to privilege richness of the information collected over the statistical power in representing patients' general positions. Based on the results of this preliminary qualitative study, a dedicated quantitative survey on European patients may provide more reliable data on patient perspectives on cell and gene therapies.

Moreover, from a methodological point of view, the choice of running all interviews in English may have affected the ability of interviewees to answer providing all the nuances they would have used in their mother tongue. To minimize this risk the guidelines of the interview was shared in advanced to allow interviewees to prepare and after the interview the transcript was shared again to allow them to check and potentially to adjust their statement. Nonetheless, the language barrier could not be completely overcome.

ATMP access and information vary from country to country in Europe, resulting a potential bias in opinion depending on the residency of the interviewees. However, the interviewees are expert patient advocates representing super-national patient organizations and have general visions, developments of their disease areas.

## Data Availability Statement

The datasets presented in this article are not readily available because due to their nature (semi-structured interviews) data cannot be fully anonymized. Requests to access the datasets should be directed to Stefano Benvenuti-sbenvenuti@telethon.it.

## Author Contributions

SBe designed the study, prepared the protocol, conducted some of the interviews, and contributed to analysing the data and to writing the manuscript. CMW contributed to writing the manuscript. SBo conduct most of the interviews, analyzed the data and contributed to writing the manuscript. All authors contributed to the article and approved the submitted version.

## Funding

This work was funded by the European Union's Horizon 2020 research and innovation program for RESTORE, under Grant Agreement No: 820292.

## Conflict of Interest

The authors declare that the research was conducted in the absence of any commercial or financial relationships that could be construed as a potential conflict of interest.

## Publisher's Note

All claims expressed in this article are solely those of the authors and do not necessarily represent those of their affiliated organizations, or those of the publisher, the editors and the reviewers. Any product that may be evaluated in this article, or claim that may be made by its manufacturer, is not guaranteed or endorsed by the publisher.

## References

[1] Cuende N Rasko JEJ Koh MBC Dominici M Ikonomou L. Cell, tissue and gene products with marketing authorization in 2018 worldwide. Cytotherapy. (2018) 20:1401–13. 10.1016/j.jcyt.2018.09.010 30366616

[2] Shukla V Seoane-Vazquez E Fawaz S Brown L Rodriguez-Monguio R. The landscape of cellular and gene therapy products: authorization, discontinuations, and cost. Human Gene Therapy Clin Dev. (2019) 30:102–13. 10.1089/humc.2018.201 30968714

[3] Pimenta C Bettiol V Alencar-Silva T Franco OL Pogue R Carvalho JL. Advanced Therapies and Regulatory Framework in Different Areas of the Globe: Past, Present, and Future. Clinical Therapeutics. Excerpta Medica Inc. (2021). Available online at: https://pubmed.ncbi.nlm.nih.gov/33892966/ (accessed May 12, 2021). 10.1016/j.clinthera.2021.02.006 33892966

[4] Rigter T Klein D Weinreich SS Cornel MC. Moving somatic gene editing to the clinic: routes to market access and reimbursement in Europe. Eur J Human Genet. (2021). Available online at: https://pubmed.ncbi.nlm.nih.gov/33850300/ (accessed May 12, 2021). 10.1038/s41431-021-00877-y PMC8484669 33850300

[5] Dunbar CE High KA Joung JK Kohn DB Ozawa K Sadelain M. Gene therapy comes of age. Science. (2018) 359:eaan4672. 10.1126/science.aan4672 29326244

[6] Yu TTL Gupta P Ronfard V Vertès AA Bayon Y. Recent progress in European advanced therapy medicinal products and beyond. Front Bioeng Biotech. (2018) 6:130. 10.3389/fbioe.2018.00130 30298129 PMC6161540

[7] European Parliament and Council of the European Union. Regulation (EC) No 1394/2007 of the European parliament and of the council of 13 November 2007 on advanced therapy medicinal products and amending Directive 2001/83/EC and Regulation (EC) No 726/2004 (Text with EEA relevance) (2007).

[8] European Parliament and Council of the European Union. Directive 2001/83/EC of the European Parliament and of the Council of 6 November 2001 on the Community Code Relating to Medicinal Products for Human Use. (2001). Available online at: https://eur-lex.europa.eu/legal-content/EN/TXT/PDF/?uri=CELEX:02001L0083-20121116&from=EN

[9] U.S. Department of Health and Human Services, Food and Drug Administration, Center for Biologics Evaluation and Research. Expedited Programs for Regenerative Medicine Therapies for Serious Conditions; Guidance for Industry. (2019). Available online at: http://www.fda.gov/BiologicsBloodVaccines/GuidanceComplianceRegulatoryInformation/Guidances/default.htm (accessed May 18, 2021).

[10] Alliance for Regenerative Medicine. Growth and Resilience in Regenerative Medicine - Annual Report 2020 (2021). Available online at: http://www.alliancerm.org (accessed May 12, 2021).

[11] Approved Cellular and Gene Therapy Products|FDA. Available online at: https://www.fda.gov/vaccines-blood-biologics/cellular-gene-therapy-products/approved-cellular-and-gene-therapy-products (accessed May 12, 2021).

[12] Abou-El-Enein M Elsanhoury A Reinke P. Overcoming challenges facing advanced therapies in the EU market. Cell Stem Cell. (2016) 19:293–7. 10.1016/j.stem.2016.08.012 27588746

[13] Trapani I Banfi S Simonelli F Surace EM Auricchio A. Gene therapy of inherited retinal degenerations: prospects and challenges. Human Gene Therapy. (2015) 26:193–200. 10.1089/hum.2015.030 25762209 PMC4410187

[14] Ylä-Herttuala S. Gene and cell therapy: success stories and future challenges. Molecular Therapy. (2019) 27:891–2. 10.1016/j.ymthe.2019.04.012 31010739 PMC6520518

[15] Gohar F Maschmeyer P Mfarrej B Lemaire M Wedderburn LR Roncarolo MG. Driving medical innovation through interdisciplinarity: unique opportunities and challenges. Front Med. (2019) 6:35. 10.3389/fmed.2019.00035 30863750 PMC6400109

[16] Heathman TR Nienow AW McCall MJ Coopman K Kara B Hewitt CJ. The translation of cell-based therapies: clinical landscape and manufacturing challenges. Regenerative Med. (2015) 10:49–64. 10.2217/rme.14.73 25562352

[17] ten Ham RMT Hoekman J Hövels AM Broekmans AW Leufkens HGM Klungel OH. Challenges in advanced therapy medicinal product development: a survey among companies in Europe. Mol Therapy-Methods Clin Dev. (2018) 11:121–30. 10.1016/j.omtm.2018.10.003 30456217 PMC6234262

[18] Varju M Sándor J. Creating European Markets Through Regulation: The Case of the Regulation on Advanced Therapy Medicinal Products. European Law Review (2016).

[19] Cuende N Boniface C Bravery C Forte M Giordano R Hildebrandt M. The puzzling situation of hospital exemption for advanced therapy medicinal products in Europe and stakeholders' concerns. Cytotherapy. (2014) 16:1597–600. 10.1016/j.jcyt.2014.08.007 25293816

[20] Ronco V Dilecce M Lanati E Canonico PL Jommi C. Price and reimbursement of advanced therapeutic medicinal products in Europe: are assessment and appraisal diverging from expert recommendations? J Pharmaceutical Policy Practice. (2021) 14:30. 10.1186/s40545-021-00311-0 33741076 PMC7980570

[21] Jönsson B Hampson G Michaels J Towse A von der Schulenburg JMG Wong O. Advanced therapy medicinal products and health technology assessment principles and practices for value-based and sustainable healthcare. Eur J Health Economics. (2019) 20:427–38. 10.1007/s10198-018-1007-x 30229376 PMC6438935

[22] Borán T Menezes-Ferreira M Reischl I Celis P Ferry N Gänsbacher B. Clinical development and commercialization of advanced therapy medicinal products in the european union: how are the product pipeline and regulatory framework evolving? Human Gene Therapy Clin Dev. (2017) 28:126–35. 10.1089/humc.2016.193 28510497

[23] Haerry D Landgraf C Warner K Hunter A Klingmann I May M. EUPATI and patients in medicines research and development: guidance for patient involvement in regulatory processes. Front Med. (2018) 5:231. 10.3389/fmed.2018.00230 30175100 PMC6107804

[24] Jannetta E. A Qualitative Study of Cystic Fibrosis (CF) Patients' Expectations of Gene Therapy. (2009). 10.1016/S1569-1993(10)60391-8

[25] Rodriguez-Monguio R Spargo T Seoane-Vazquez E. Ethical imperatives of timely access to orphan drugs: is possible to reconcile economic incentives and patients' health needs? Orphanet J Rare Dis. (2017) 12:1. 10.1186/s13023-016-0551-7 28057032 PMC5217554

[26] Sacristán JA Aguarón A Avendaño-Solá C Garrido P Carrión J Gutiérrez A. Patient involvement in clinical research: why, when, and how. Patient Preference Adherence. (2016) 10:631–40. 10.2147/PPA.S104259 27175063 PMC4854260

[27] du Plessis D Sake JK Halling K Morgan J Georgieva A Bertelsen N. Patient centricity and pharmaceutical companies: is it feasible? Therapeutic Innovation Regulatory Sci. (2017) 51:460–7. 10.1177/2168479017696268 30227057

[28] Battaglia M Furlong P Wulffraat NM Bellutti Enders F. Improving the translational medicine process: moving patients from “End-Users” to “Engaged Collaborators.” *Front Med*. (2019) 6:110. 10.3389/fmed.2019.00110 31165071 PMC6536600

[29] Holmes L Cresswell K Williams S Parsons S Keane A Wilson C. Innovating public engagement and patient involvement through strategic collaboration and practice. Res Involvement Engage. (2019) 5:30. 10.1186/s40900-019-0160-4 31646001 PMC6802177

[30] Lee Aiyegbusi O Macpherson K Elston L Myles S Washington J Sungum N. Patient and public perspectives on cell and gene therapies: a systematic review.*Nat Commun*. (2020) 11:6265. 10.1038/s41467-020-20096-1 33293538 PMC7722871

[31] Salzman R Cook F Hunt T Malech HL Reilly P Foss-Campbell B. Addressing the value of gene therapy and enhancing patient access to transformative treatments. Mol Ther. (2018) 26:2717–26. 10.1016/j.ymthe.2018.10.017 30414722 PMC6277509

[32] Hollak CEM Sirrs S van den Berg S van der Wel V Langeveld M Dekker H. Registries for orphan drugs: generating evidence or marketing tools? Orphanet J Rare Dis. (2020) 15. Available online at: https://pubmed.ncbi.nlm.nih.gov/32883346/ (accessed October 14). 10.1186/s13023-020-01519-0 PMC7469301 32883346

[33] Cave A Kurz X Arlett P. Real-World data for regulatory decision making: challenges and possible solutions for Europe. Clin Pharmacol Therapeut. (2019) 106:36–9. Available from: www.cpt-journal.com (accessed May 12, 2021). 10.1002/cpt.1426 30970161 PMC6617710

[34] Bauer G Elsallab M Abou-El-Enein M. Concise review: a comprehensive analysis of reported adverse events in patients receiving unproven stem cell-based interventions. Stem Cells Translational Med. (2018) 7:676–85. 10.1002/sctm.17-0282 30063299 PMC6127222

[35] Peay HL Fischer R Tzeng JP Hesterlee SE Morris C Martin AS. Gene therapy as a potential therapeutic option for Duchenne muscular dystrophy: a qualitative preference study of patients and parents. PLoS ONE. (2019) 14:e0213649. 10.1371/journal.pone.0213649 31042754 PMC6493713

[36] Cardano M. La ricerca qualitativa. Bologna: Il Mulino (2011).

[37] Green J Thorogood N. Qualitative Methods for Health Research. 4th Edn. London: SAGE Publications Inc. (2004).

[38] Chatham House Rule. Chatham House – International Affairs Think Tank. Available online at: https://www.chathamhouse.org/about-us/chatham-house-rule (accessed May 18, 2021).

[39] Jørgensen J Kefalas P. Reimbursement of licensed cell and gene therapies across the major European healthcare markets. J Market Access Health Policy. (2015) 3:29321. 10.3402/jmahp.v3.29321 27123175 PMC4802687

[40] Jørgensen J Hanna E Kefalas P. Outcomes-based reimbursement for gene therapies in practice: the experience of recently launched CAR-T cell therapies in major European countries. Eur Countries J Market Access Health Policy. 8. 10.1080/20016689.2020.1715536 32082514 PMC7006635

[41] Vellekoop H Huygens· S Matthijs V Szilberhorn L Tamás Z Nagy B. Guidance for the harmonisation and improvement of economic evaluations of personalised medicine on behalf of the HEcoPerMed consortium. Pharmaco Economics. (2021) 39:771–88. 10.1007/s40273-021-01010-z 33860928 PMC8200346

[42] Autorisations temporaires d'utilisation (ATU) - Ministère des Solidarités et de la Santé [Internet]. Available online at: https://solidarites-sante.gouv.fr/soins-et-maladies/medicaments/professionnels-de-sante/autorisation-de-mise-sur-le-marche/article/autorisations-temporaires-d-utilisation-atu (accessed May 18, 2021).

[43] European Medicines Agency. EMA Warns Against Using Unproven Cell-Based Therapies. (2020). Available online at: http://www.ema.europa.eu/contact (accessed May 12, 2021).

[44] Spindler P Lima BS. Editorial: The European Patients Academy on Therapeutic Innovation (EUPATI) Guidelines on patient involvement in research and development. Front Med. (2018) 5:310. 10.3389/fmed.2018.00310 30467543 PMC6236003

[45] National Academies of Sciences E. Human Genome Editing: Science, Ethics, and Governance. Washington, DC: National Academies Press (2017). p. 1–310. 28796468

[46] Cwik B. Moving beyond “therapy” and “enhancement” in the ethics of gene editing. Cambridge Quarterly Healthcare Ethics. (2019) 28:695–707. 10.1017/S0963180119000641 31526421 PMC6751566

[47] Howard HC van El CG Forzano F Radojkovic D Rial-Sebbag E de Wert G. One small edit for humans, one giant edit for humankind? Points and questions to consider for a responsible way forward for gene editing in humans. Eur J Human Genet. (2018) 26:1–11. 10.1038/s41431-017-0024-z 29192152 PMC5839051

[48] Niemiec E Howard HC. Ethical issues related to research on genome editing in human embryos. Computat Structural Biotech J. (2020) 18:887–96. 10.1016/j.csbj.2020.03.014 32322370 PMC7163211

[49] Critchley C Nicol D Bruce G Walshe J Treleaven T Tuch B. Predicting public attitudes toward gene editing of germlines: the impact of moral and hereditary concern in human and animal applications. Front Genet. (2019) 10:704. 10.3389/fgene.2018.00704 30687386 PMC6334182

[50] de Wert G Heindryckx B Pennings G Clarke A Eichenlaub-Ritter U van El CG. Responsible innovation in human germline gene editing: Background document to the recommendations of ESHG and ESHRE. Eur J Human Genet. (2018) 26:450–70. 10.1038/s41431-017-0077-z 29326429 PMC5891502

