# Supplementary material for: Perspectives, Expectations, and Concerns of European Patient Advocates on Advanced Therapy Medicinal Products
Source: Front Med (Lausanne). 2021 Nov 23;8:728529. doi: 10.3389/fmed.2021.728529 (PMC8649896; doi:10.3389/fmed.2021.728529)
Supplement: Supplementary file 1 [file Data_Sheet_1.PDF]

Health by Advanced Therapies

Patients view on Advanced Therapies

Semi-structured interviews with patient advocates

This project has received funding from the European Union's Horizon 2020 research and innovation programme under grant agreement No 820292.

1. Purpose

In the frame of the RESTORE project, we aim at collecting the view of patient advocates on some hot topics in the field of Advanced Therapy Medicinal Products (ATMPs) to prepare a report (public deliverable of the project) presenting it.

2. Methods

We will run a dozen of semi-structured interviews with representatives of key umbrella and patient organizations at European level. Each interview will last 30 to 45 minutes and will be recorded. However we will keep the recording confidential and any disclosure of the opinions expressed will go under the so-called Chatham House Rule (https://www.chathamhouse.org/chatham-house-rule); therefore, the points of view expressed during the interview could be included in the final report but without identifying, either explicitly or implicitly, who said what.

3. Guidelines for interview

Here below the set of topics for discussion during the interview:

1. Are Advanced Therapy Medicinal Products (ATMPs)[1] a hot topic for patients or not?
   a. If yes, is this always true (even when “good enough” chronic treatments are available) or is this interested limited to certain specific diseases/group of diseases?
2. Access – apart from the pricing, access to ATMPs is currently limited also by the long and complex procedure for the qualification of centres that can administer those therapies:
   a. Which model do you think would be preferable – a model with several centres in every country, geographically distributed and treating a relatively low number of patients (mainly local patients) or the creation/recognition of European clinical hubs for ATMPs treating an high number of patients coming from all around the EU?
   b. In case of clinical hubs, do you think patient cross-border mobility may be a major obstacle for access? Do you have any solution in mind?
3. Pricing
   a. Do you think high prices are a problem? What is your opinion on managed entry agreement[2] / value-based pricing?
   b. What does value mean for you (e.g. key treatment endpoints (i.e., efficacy and/or benefit), duration of the effect, safety, and cost[3])? Should Patients Centred Outcome Measures (PCOMs)[4] and/or patients perceived/reported value be part of the deal?

---

[1] ATMPs are are medicines for human use that are based on genes, tissues or cells. They offer groundbreaking new opportunities for the treatment of disease and injury. ATMPs can be classified into three main types: gene therapy medicines; somatic-cell therapy medicines; tissue-engineered medicines. (source: EMA)

[2] These schemes are agreements between manufacturers and decision-making bodies designed to reduce the risk incurred by health services in reimbursing new medicines when, at the point of price negotiation, there is still great uncertainty regarding the clinical and cost effectiveness of the new product. These schemes allow the development of further evidence or entail a risk-sharing component to mitigate the risk for health services of paying for a non-cost-effective treatment. (source: Grimm et al. 2017, PMID: 28849538)

[3] Salzman et al. 2018, PMID: 30414722.

[4] PCOMs are clinical endpoints (measures) that allow understanding the impact of the disease on the people who have it, and what patients value most in terms of alleviation. PCOMs should measure the right outcomes

4. Value based pricing depends on the collection of long-term follow-up data:
    a. Do you think this is feasible on a relatively large scale and for a long period (e.g. 36 months after treatment, taking also into account that the patient may be fully healed)?
    b. Do you think patients' organization could play a role/commit to avoid patients drop-out from long-term post-treatment data collection? If a patients receives an expensive treatment (ATMP) for free (fully reimbursed) is it acceptable for the patient to be obliged to enrol in such follow-up studies?
    c. Who should pay for the infrastructure for data collection?
    d. Are you concerned about data sharing?
5. Access pathways for ATMPs (clinical trial VS market authorized VS hospital exemption VS unauthorized treatments): preferences, awareness and ideas – for example, should a patient go for a first generation marketed product or should he/she choose a clinical trial of a second-generation product (considering that choosing one prevent from choosing the other)? Is the approval process (both for Clinical Trials and for Marketing Authorization) and the price negotiation quick enough? Would you prefer a less regulated process to improve the speed of approval?
6. Concerns about Safety – what is the patients perception on this, is hope to get a cure/treatment stronger than fear for adverse events?
    a. If yes and hope is stronger, do you think this could play a role in the diffusion of unauthorized treatments?
7. Ethical concerns – broadly speaking, what are your personal concerns about gene editing techniques: ethical boundaries, risks, disillusion, etc.
8. Education – Cell and gene therapies are already in use by the treatment centers in the EU and the amount of authorised ATMPs are projected to increase rapidly over the next 10 years. In this context:
    a. do you think there is a need for educational tools for patients, healthcare professionals (or for both groups)? others?
    b. What shall be the focus of a training: product development and patient engagement, clinical trials and patient selection, safety issue, access - including the reimbursement models, life after therapy. Would you like to learn from other patient's journeys?
    c. What would you consider as the most efficient training tools for patients (webinars with ATMPs experts, dedicated website for patients, newsletters, seminars, visit of different manufacturing facilities, other tools)?

(that resonate with patients' daily experience of the disease, preferences, expectations and values) in the right patients (i.e. across a continuum of disease severity and manifestations). (Morel et at. 2017, PMID: 29096663).

4. Informed Consent

Consent for participation in a Key Opinion Leader Interview for the RESTORE Project

Part I: Information Sheet

Overview and Purpose

The primary objective of this interview is to gather the input of patients advocates on a list of hot topics in the field of Advanced Therapy Medicinal Products (ATMPs) to prepare a report (public deliverable of the project) presenting it.

Participant Selection

You are being invited to take part in this interview research because we feel that your experience can contribute to describe the view of patients on the proposed topics.

Voluntary Participation

Your participation in this interview is voluntary. It is your choice whether to participate or not. If you choose not to participate today, your potential participation in any future research with the organizers or funders of this study will not be excluded.

Procedures

Brief introduction to the format of the research project.

We are inviting you to take part in this interview. If you accept, you will be asked to participate in a recorded phone/conferencing system or face-to-face interview that will be scheduled at your convenience.

During the interview, you will be asked about your views on past current and future trends in rare disease policy. If you do not wish to answer any of the questions during the interview, you may say so and the interviewer will move on to the next question. No one else but the interviewer will be present during the interview unless you would like someone else to be there.

Duration

The interview will last for approximately one 45 minutes. You may need to spend additional time (max 30 minutes) for interview preparation or for brief follow-up email contact should the interviewer need any clarifications following the interview.

Risks

We are asking you to share with us professional experience and opinions. You do not have to answer any question or take part in the interview so feel free not to do it. You do not have to give us any reason for not responding to any question, or for refusing to take part in the interview.

Benefits

There will be no direct benefit to you, but your participation will help us to better understand the view of patients on ATMPs and to ultimately incorporate such a view in the strategic research roadmap that the RESTORE project is expected to deliver.

Confidentiality

The information recorded is confidential until you have approved all summaries of the interview. A metanalysis of qualitative data collected during these interviews will be part of a final public report. You will have the right to withdraw your recorded interview until the end of the interview period (March 2020). All data related to the study (informed consent forms, interview recording and products of analysis) will be kept on a secure server at Fondazione Telethon. In any case, even when reporting quotation of your interview, the interviewer(s) will make any reasonable effort to avoid that you can be directly identified by the readers. The full list of interviewed experts may be published but no direct link with the content of the interview will be disclosed.

Sharing the Results

The knowledge that we get from this research will be shared with you and your community. Any results related to this study may not be published without prior approval from the project partners.

Right to Refuse or Withdraw

You do not have to take part in this interview if you do not wish to do so, and choosing to participate will not affect any potential future collaboration with RESTORE partners in any way. You may stop participating in the interview at any time that you wish without any reason.

Who to Contact

If you have any questions, you can ask them now or later. If you wish to ask questions later, you may contact any of the following:

Stefano Benvenuti
Fondazione Telethon
+39 02 XXXX

Chiuhui Mary Wang
Fondazione Telethon
+39 02 XXX

Part II: Certificate of Consent

I have been given sufficient information about this project and the purpose of my participation as an interviewee has been explained to me and is clear.
My participation as an interviewee in this project is voluntary. I allow the interviewer(s) to take written notes during the interview. I also allow the audio/video recording of the interview. It is clear to me that in case I do not want the interview to be recorded I am at any point during my interview fully entitled to withdraw from participation.
I have the right not to answer any of the questions. If I feel uncomfortable in any way during the interview session, I have the right to withdraw from the interview.
I have been given the explicit guarantees that, if I wish so, I can ask that my recorded interview be withdrawn from the project until the end of the study period (March 2020).
I understand the results of all or any part of this interview can be published any time in the future; in case the reporting of my view could led to my direct identification, I will be contacted for explicit consent. In all cases subsequent uses of records and data will be subject to the applicable laws and internal policies.

I confirm that I have received the attached protocol and agree to participate in the interview in accordance with the contents, and I understand and agree to the terms described.

Signature: ___________________

Print Name: __________________

Date: _______________________
